# Isolation of anticancer constituents from flos genkwa (*Daphne genkwa* Sieb.et Zucc.) through bioassay-guided procedures

**DOI:** 10.1186/1752-153X-7-159

**Published:** 2013-09-23

**Authors:** Simeng Li, Guixin Chou, Youcheng Hseu, Hsinling Yang, HiuYee Kwan, Zhiling Yu

**Affiliations:** 1 The MOE Key Laboratory for Standardization of Chinese Medicines, Institute of Chinese Materia Medica, Shanghai University of Traditional Chinese Medicine, Cai Lun Road 1200, Zhangjiang Hi-Tech Park, Shanghai 201210, PR China; 2 Department of Cosmeceutics, China Medical University, Taichung 40402, Taiwan; 3 Institute of Nutrition, China Medical University, Taichung, Taiwan; 4 Shanghai R&D Center for Standardization of Chinese Medicines, Shanghai 201203, PR China; 5 School of Chinese Medicine, Hong Kong Baptist University, Kowloon Tong, Hong Kong, China

**Keywords:** Daphne genkwa, Antitumor, MTT, Flow cytometric, Western blot

## Abstract

**Background:**

Flos Genkwa (yuanhua in Chinese), the dried flower buds of *Daphne genkwa* Sieb.et Zucc. (Thymelaeaceae), is a traditional Chinese medicinal herb mainly used for diuretic, antitussive, expectorant, and anticancer effects. However, systematic and comprehensive studies on Flos Genkwa and its bioactivity are limited.

**Results:**

After confirmation of the anti-tumor activity, the 95% ethanolic extract was subjected to successive solvent partitioning to petroleum ether, dichloromethane, n-butanol, and water soluble fractions. Each fraction was tested using the same biological activity model, and the dichloromethane fraction had the highest activity. The dichloromethane fraction was subjected to further chromatographic separation for the isolation of compounds **1–13**. Among the 13 compounds, the diterpene esters (compounds **10**–**13**) showed anticancer activity, whereas the flavonoids, lignanoids, and peptides showed moderate activity. Compound **13** was a new daphnane diterpenoid, which was named genkwanin VIII.

The preliminary antitumor mechanism of yuanhuacine was studied by protein expression and cell cycle analysis in MCF-7 cancer cells.

**Conclusion:**

The present investigation tends to support the traditional use of Flos Genkwa for treating cancer. Through bioassay-guided fractionation and isolation techniques, the CH_2_Cl_2_ fraction was determined as the active fraction of the flower buds of *D. genkwa*, and the anti-tumor activity was ascribable to the compounds **10–13**.

## Background

Flos Genkwa, the dried flower buds of *Daphne genkwa*, is a commonly used traditional Chinese medicine (TCM) known to have purgative and diuretic effects, which helps to remove fluid retention [1]. Flos Genkwa is also used as abortifacient [2] and expectorant [3]. As a traditional Chinese toxic herb, this medicine possesses anticancer effect [4-6], based on an important proposition of TCM theory “Fighting fire with fire” (Poisonous Chinese herbal medicine for cancer therapy). In clinical applications, this TCM has been used as “Shi-Zao-Tang” to treat malignant ascites [7,8], as “San-Leng-Jian-Wan” to treat tumor models of human ovarian cancer [9], and in a sticking-plaster formula to treat malignant pleural effusion, malignant ascites, and icterus [10]. The material basis of the antitumor activity needs to be investigated. Various flavonoids, daphnane-type diterpene ester [11,12], lignanoid, coumarins, and amides [13,14] have been identified in *D. genkwa*. Recent studies have reported that total flavonoids inhibited the growth of Lewis lung carcinoma in C57BL6 mice [15]. (-)-Syringaresinol potently inhibited the proliferation of human promyelocytic HL-60 cells through G1 arrest and apoptosis induction [16]. Daphnoretin caused human osteosarcoma (HOS ) cell cycle arrest in the G2/M phase and triggered the caspase-3-dependent apoptotic pathway [17]. Yuanhuadine inhibited growth against human lung cancer cells and cell cycle arrest in the G0/G1 and G2/GM phases with the up-regulation of p21 and the down-regulation of cyclins, CDK2, CDK4, and c-Myc expressions [18]. However, thus far the systematic research on the constituents responsible for the anticancer activity of the plant remains unclear. Thus, the potential antitumor chemotherapeutic activity of Flos Genkwa needs to be ascertained.

In the present study, the anticancer ability of the extracts, fractions, and compounds obtained through activity-guided fractionation were studied *in vivo* and *in vitro*, to elucidate the role of anticancer compounds in this plant. The chemical structures were established using NMR spectroscopic, mass-spectral analyses, and published data. Thirteen compounds were isolated from the dichloromethane fraction, which had the highest activity, and their structures were elucidated to be daphnoretin (**1**) [19], genkwanin (**2**), 3′-hydroxygenkwanin (**3**) [20], apigenin (**4**) [21], tiliroside (**5**) [22], pinoresinol (**6**) [23], syringaresinol (**7**) [24], lariciresinol (**8**) [25], aurantiamide acetate (**9**) [26], yuanhuacine (**10**), yuanhuadine (**11**) [4], genkwanine III (**12**) [27], and genkwanine VIII (**13**). The inhibitory activities of these compounds were investigated on nine tumor cells *in vitro* by MTT assay, and the results indicated that daphnane-type diterpene esters (**10**–**13**) showed significant antitumor activity (Table 1). To the best of our knowledge, this is the first report on the isolation of cytotoxic compounds from *D. genkwa* against MCF-7, HT-29, MDA-MB-231, Huh 7, Colo 205, AGS, A 2058, and B 16 cell lines. Yuanhuacine was one of the compounds that exhibited potent cytotoxic activities. Furthermore, the mechanism of action of yuanhuacine against MCF-7 was studied using flow cytometry and Western blot analysis.

**Table 1:** Cytotoxic effects of the tested compounds

	**Huh 7**	**A 2058**	**B 16**	**231**	**MCF-7**	**COLO 205**	**A 549**	**AGS**	**HT-29**
	**IC**_**50 **_**μg/mL**								
Yuanhuacine	>60	15	18	20	15	3	>60	17	23
Genkwanine III	>60	54	>60	>60	>60	>60	34	>60	>60
Genkwanine VIII	>60	58	>60	>60	48	34	>60	12	29
Yuanhuadine	13	12	11	19	10	2	3	16	13
Daphnoretin	48	17	13	>60	>60	>60	>60	34	>60
Genkwanin	56	>60	–	>60	>60	>60	–	>60	>60
3*'*-hydroxygenkwanin	>60	21	–	>60	40	>60	–	43	>60
Tiliroside	>60	>60	–	>60	43	>60	–	>60	>60
Apigenin	>60	>60	–	>60	>60	>60	–	>60	>60
Pinoresinol	>60	>60	–	>60	>60	>60	–	>60	>60
Syringaresinol	>60	>60	–	>60	>60	57	–	>60	>60
Lariciresinol	>60	>60	–	>60	>60	>60	–	>60	>60
Aurantiamide acetate	>60	>60	–	56	>60	>60	–	>60	>60

Table 1: Inhibitory effects of the compounds from dichloromethane fraction of the Flos Genkwa on the proliferation of MDA-MB-231, Huh 7, AGS, Colo 205, MCF-7, HT-29, A 549, A 2058 and B 16 cells. Cells were treated with 0–20 μg/mL of extracts for 24 h.

## Results and discussion

### Isolation and identification

The dried flower buds of *D. genkwa* (20.0 kg) were extracted with 9% ethanol extensively at room temperature and evaporated to dryness to yield a dark crude ethanol extract (2 kg). This extract was then dissolved in water to form a suspension, partitioned with petroleum ether (20 L×3 times), dichloromethane (20 L×3 times), n-butanol (20 L×3 times), respectively, and then evaporated under reduced pressure at 60°C to yield the petroleum fraction (346 g), dichloromethane fraction (277 g), n-butanol fraction (532 g), and water fraction (489 g).

For the MTT assay, an S180 tumor model was used to evaluate their antitumor agents. The results suggested that the antitumor agents were mainly contained in the dichloromethane fraction. Further experiments were performed on the dichloromethane extract to isolate the antitumor compounds. The dichloromethane fraction was subjected to column chromatography to yield 10 fractions. An activity-directed isolation process was used to obtain the compounds, and 12 known compounds with one new compound (genkwanin VIII) were isolated from this fraction.

The new structure was elucidated by modern spectroscopic techniques including ^1^H-NMR (500 MHz), ^13^C-NMR (125 MHz), 2D-NMR, and MS. The known compounds were identified by comparing their ^1^H-NMR (400/500 MHz) and ^13^C-NMR (100/125 MHz) data with those of authentic samples or with those reported in literatures. The purity of each compound exceeded %.

**Figure 1:**
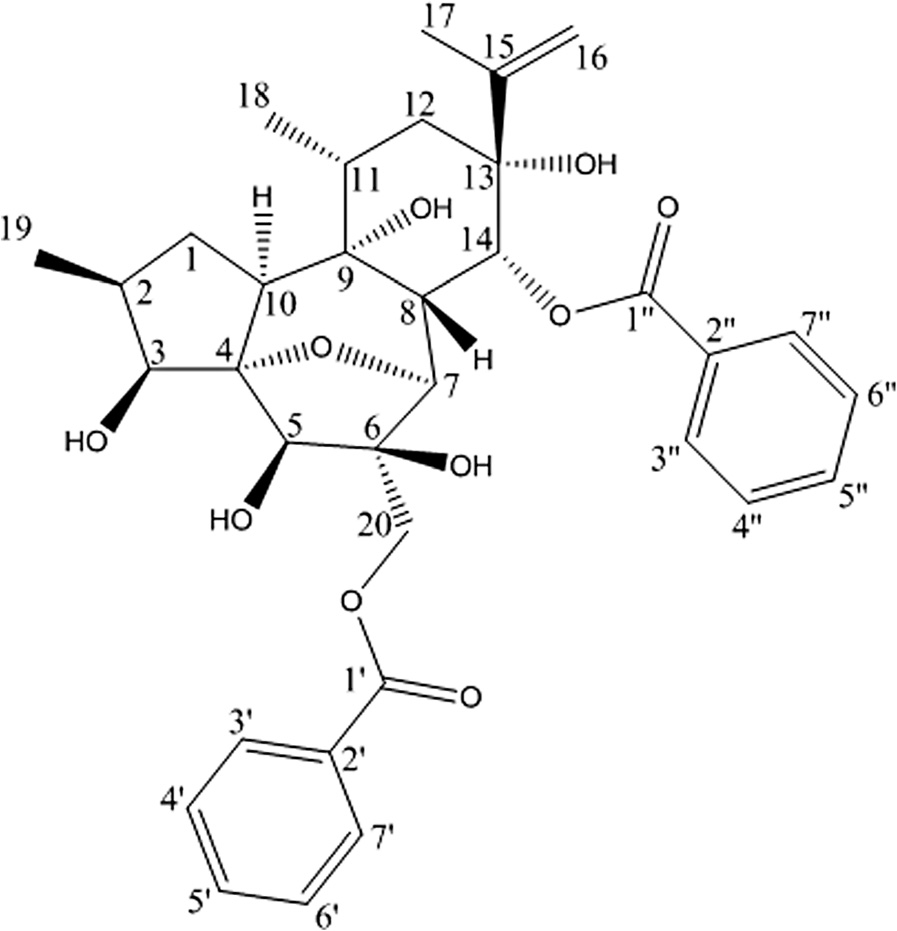
Molecular structure of genkwanine VIII (13).

Genkwanin VIII (**13**): White amorphous, its molecular formula was assigned to be C_34_H_40_O_10_, according to its HREIMS (*m/z* 631.2521 [M+Na]^+^, calcd. for C_34_H_40_O_10_Na, 631.2519) and NMR results. Analysis of its ^13^C NMR and DEPT spectra (Table 2) established the presence of 34 carbon resonances, including 3 methyls, 4 methylenes (1 oxygenated), 18 methines (4 oxygenated), and 9 quaternary carbons (2 carbonyls and 4 oxygenated). The ^1^H and ^13^C NMR spectroscopic data (Table 2) of compound **13** were similar to those of genkwanin III [15], a daphnane-type diterpene ester, except for the additional benzoyl group, with signals at δ_H_ 7.91 (2H, d, *J*=7.5 Hz, H-3*"*, 7*"*), 7.51(2H, t, *J*=7.5 Hz, H-4*"*, 6*"*), and 7.48(1H, m, H-5*"*); δ_C_ 166.4 (C-1*"*), 129.7(C-2*"*), 129.6 (C-3*"*, 7*"*), 128.3 (C-4*"*, 6*"*), and 133.0 C-5*"*). In the ^1^H-NMR spectrum of **13**, the methine proton signal of H-14 shift downfield from δ_H_ 4.1 (1H, brs) to 5.92 (1H, s), indicating that the benzoyl moiety was located at C-14. This result was further confirmed by the HMBC experiment for **13**. The key correlation from H-14 to C-1″ was observed. The configuration of **13** was elucidated by NOESY experiment (Table. 1) and was found to be the same as those of genkwanin III with α-orientations of H-2, H-10, 9-OH, 13-OH, and O-4, 14, and β-orientations of 3-OH, 5-OH, H-7, and H-8. Thus, compound **13** was assigned as shown in Figure 1 and named genkwanine VIII.

**Table 2:** NMR spectroscopic data of genkwanin VIII

**Position**	**δH(J in Hz)**	**δC**	**HMBC(**^**1**^**H to **^**13**^**C)**	**NOSEY**
1	1.27(1H, m)	34.9	2, 10, 19	
	1.96(1H, m)			
2	2.31(1H, m)	31.9		
3	4.23(1H, d, J=10 Hz)	74.2		2
4		90.2		
5	4.74(1H, s)	84.3		
6		83.2		
7	4.28(1H, s)	80.4	4, 5, 9, 20	8
8	2.56(1H, s)	45.4	6, 7, 9	1, 17
9		72.3		
10	1.99(1H, m)	48.9	1, 4, 9	
11	1.54(1H, m)	35.5		
12	1.76(1H, m)	35.3		
	2.09(1H, m)			
13		74.6		
14	5.92(1H, s)	72.2	7, 8, 9, 12, 13, 15, 1″	7, 8, 16, 17
15		145.3		
16	5.18(1H, s)	114.5	13, 15, 17	8, 11, 12, 17
	5.17(1H, s)			
17	1.91(3H, s)	19.2	13, 15, 16	
18	0.92(3H, d, J=6.56 Hz)	13.5	9, 12	
19	0.97(3H, d, J=7.12 Hz)	15.9	1, 2, 3	
20	4.58(1H, d, J=12)	67.2	6, 7, 1´	
	4.22(1H, d, J=12)			
1´		167.5		
2´		129.7		
3´7´	8.06(2H, d, J=8 Hz)	130.0		
4´6´	7.37(2H, m)	128.5		
5´	7.61(1H, t, J=7.5 Hz),	133.2		
1″		166.4		
2″		129.7		
3″7″	7.91(2H, d, J=7.5 Hz)	129.6		
4″6″	7.51(2H, t, J=7.5 Hz)	128.3		
5″	7.48(1H, m)	133.0		

Table 2: All values given in ppm downfield from TMS were determined in CDCl_3_ at ^1^H-NMR (400 MHz), ^13^C-NMR (100 MHz).

### Biological evaluation

**Figure 2:**
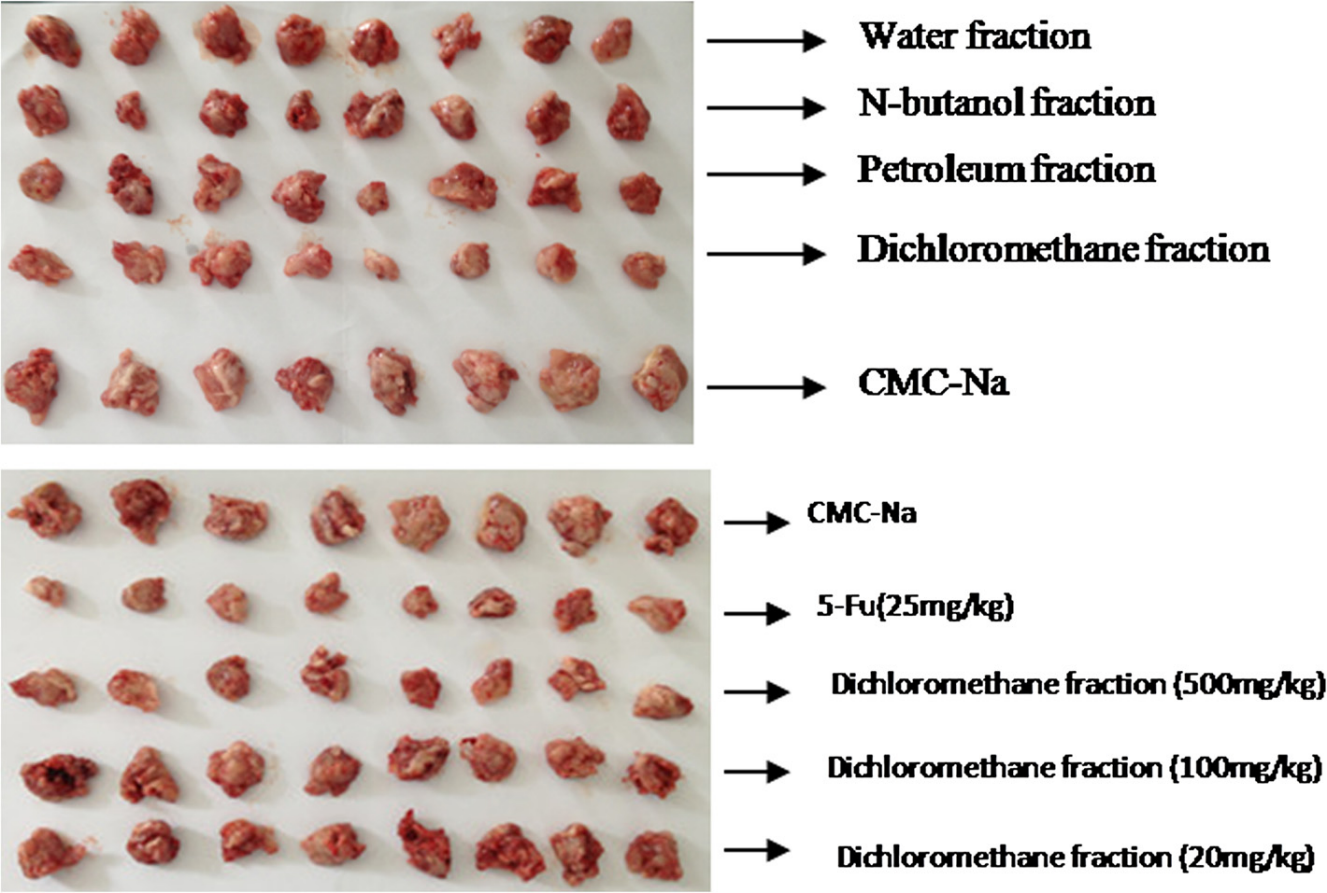
Effect of the fractions on mice transplanted with solid tumors.

The potential effect of the Flos Genkwa extracts were investigated on the viability of MCF-7, HT-29, MDA-MB-231, Huh 7, Colo 205, A549, AGS, A 2058, and B 16 cells using MTT assay with DMSO as the negative control. Cell cytotoxicity was tested in the concentration range of 0 μg/mL to 50 μg/mL (Table 3) for each extract. The results showed that the petroleum ether fraction exhibits potential cytotoxic effects in the A 2058, Colo 205, and AGS cell lines at the IC_50_ levels of 89 μg/mL to 306 μg/mL, and the dichloromethane fraction possesses potential cytotoxic effects in the A 2058, B 16, MCF-7, Colo 205, and HT 29 cell lines at the IC_50_ levels of 50 μg/mL to 289 μg/mL. The inhibition of extracts on the growth of transplanted mouse sarcoma S180 in mice is shown in Figure 2. Compared with the CMC-Na group, the dichloromethane fraction (500 mg/kg) significantly (P<0.05) inhibited the growth of S180 solid tumors, whereas other fractions (500 mg/kg) had no apparent effect. These results suggested that most of the antitumor constituents were in the dichloromethane fraction.

**Table 3:** Cytotoxic effects of the tested extracts

	**Huh 7**	**A 2058**	**B 16**	**231**	**MCF-7**	**COLO 205**	**A 549**	**AGS**	**HT-29**
	**IC**_**50 **_**μg/mL**								
Crude extract	>500	97	>500	>500	>500	>500	260	>500	>500
Petroleum ether	>500	89	>500	>500	>500	306	>500	217	>500
Dichloromethane	>500	50	98	>500	260	289	>500	>500	50
n-butanol	>500	>500	>500	>500	>500	>500	>500	>500	>500
Water	>500	>500	>500	>500	>500	431	>500	>500	>500

Inhibitory effects of the extracts from the Flos Genkwa on the proliferation of MDA-MB-231, Huh 7, AGS, Colo 205, MCF-7, HT-29, A 549, A 2058 and B 16 cells. Cells were treated with 0–50 μg/mL of extracts for 24 h.

Compounds isolated from the dichloromethane fraction of Flos Genkwa were tested for cytotoxicity in the nine cancer cell lines for 24 h. Considering that a compound usually exhibits better efficacy than an extract, each single compound was assessed at the dose range 0 μg/mL to 20 μg/mL. The results showed that daph-nane-type diterpene esters (yuanhuacine, yuanhuadine, genkwanine III, and genkwanine VIII) exhibited strong cytotoxic effects in these cell lines at the IC_50_ levels ranging from 2 μg/mL to 58 μg/mL than the other compounds. Meanwhile, six of the compounds (daphnoretin, genkwanin, 3′-hydroxygenkwanin, tiliroside, syringaresinol, and aurantiamide acetate) showed moderate IC_50_ values in six cancer cell lines (Table 1). Results suggested that daphnane-type diterpene esters are important constituents in the extract responsible for the anti-cancer activity of Flos Genkwa.

**Figure 3:**
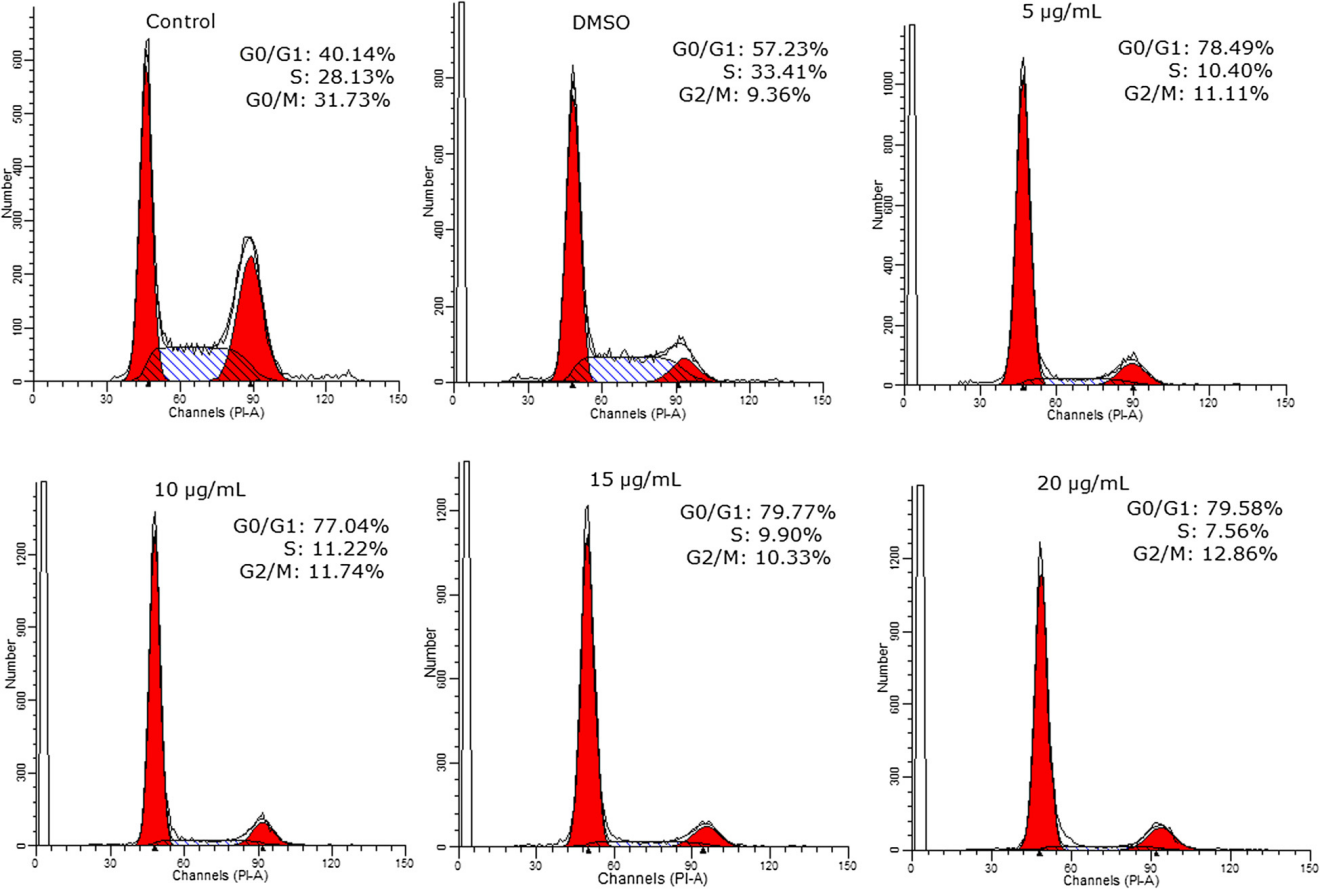
Flow cytometric analysis of yuanhuacine.

**Figure 4:**
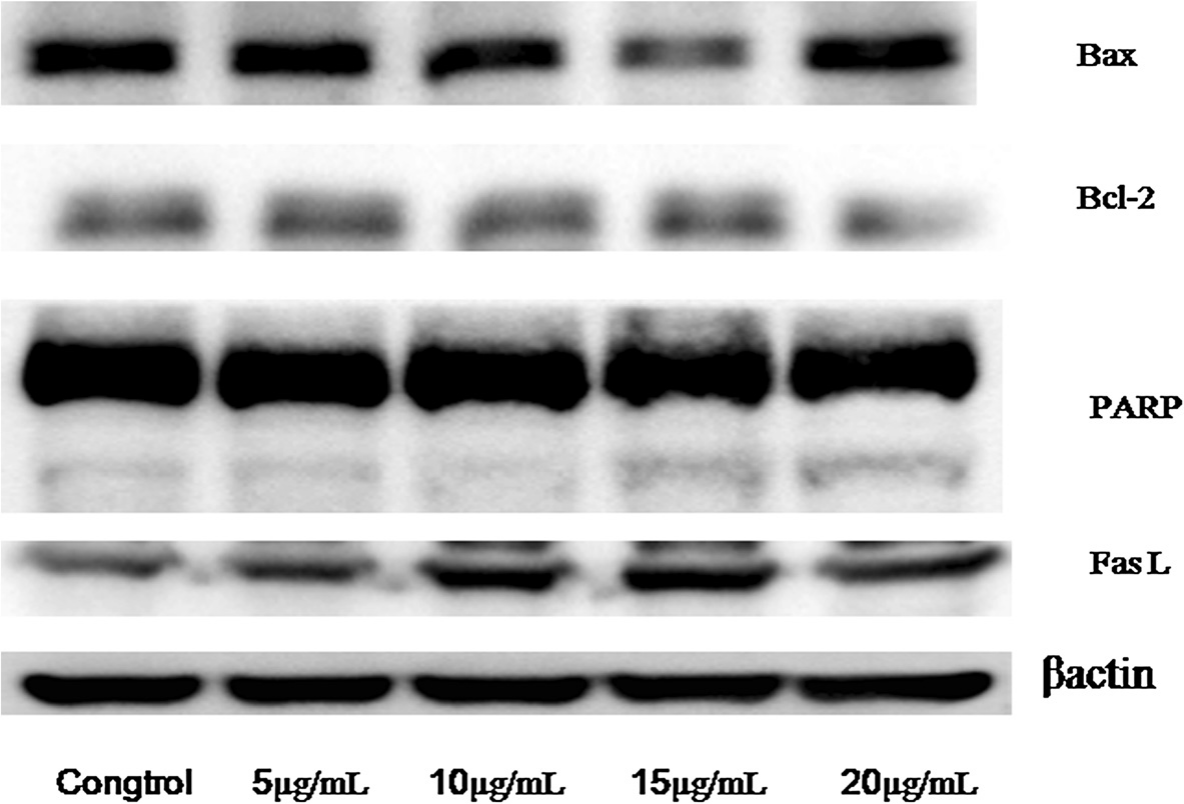
Effect of yuanhuacine on protein expressions in MCF7 cells.

Yuanhuacine was investigated to further elucidate the preliminary mechanism of daphnane-type diterpene esters and to characterize its chemotherapeutic potential effect. Yuanhuacine possessed the significant cytotoxic effect in the MCF7 cell line. To determine whether the cytotoxicity activity of yuanhuacine is related to the induction of apoptosis, the apoptosis ratios in tumor cells were quantitatively assessed by flow cytometric analysis. As a result, a special DNA peak that is usually called the sub-G1 peak or apoptotic peak appeared. As shown in Figure 3, the apoptotic peak of the yuanhuacine-treated MCF-7 cells was significantly higher than that of the control cells, suggesting that yuanhuacine induces apoptosis in MCF-7 cells. The effect of yuanhuacine on the expression in MCF-7 cells was measured by Western blot analysis (Figure 4). MCF-7 cells were treated with 0 μg/mL to 20 μg/mL of yuanhuacine for 24 h. The Bcl-2 family plays a crucial role in apoptosis because it includes both anti-apoptotic members such as Bcl-2 and pro-apoptotic members such as Bax. Expression of the anti-apoptotic protein Bcl-2 decreased, whereas Bax and cleavage of PARP expression increased after treatment with yuanhuacine. Moreover, FasL expression also increased. The results suggest that yuanhuacine induced apoptosis via the regulation of multiple signaling pathways toward an increment of death signals.

## Conclusion

In this study, an activity-directed fraction and purification process were used to isolate antitumor compounds from the flower buds of *D. genkwa*. The dichloromethane fraction exhibited good antitumor activities, consequently leading to the isolation of 13 compounds identified as daphnoretin (**1**), genkwanin (**2**), 3′-hydroxygenkwanin (**3**), apigenin (**4**), tiliroside (**5**), pinoresinol (**6**), syringaresinol (**7**), lariciresinol (**8**), aurantiamide acetate (**9**), yuanhuacine (**10**), yuanhuadine (**11**), genkwanine III (**12**), and genkwanine VIII (**13**). Genkwanin VIII was found to be a new compound. Through the MTT anti-cancer activity assay, daphnane diterpene esters showed stimulatory activity. The results suggested that daphnane-type diterpene esters may be the dominant constituents of antitumor activity in Flos Genkwa. This is the first systematic study of extracts and compounds from *D. genkwa* guided by the anti-tumor activity screen of tumor cell lines and S180 tumor models. Yuanhuacine was selected for the preliminary mechanism in the MCF-7 cell line. Results showed that yuanhuacine induced apoptosis via the regulation of multiple signaling pathways toward an increment of death signals.

A material basis for the anti-cancer effect of Flos Genkwa is provided and the scientific basis for the further investigation of these daphnane-type diterpene esters on their antitumor activities is strengthened.

## Experimental

### General procedures and reagents

^1^H-NMR, ^13^C-NMR, and 2D-NMR were recorded using a Bruker spectrometer at 25°C, with tetramethylsilane as the internal standard and CDCl_3_, DMSO-d_6_, or CD_3_OD as the solvent. Colum chromatography was performed using silica gel (100 to 200 mesh) (Qingdao Marine Chemistry Co., Qingdao, China), Sephadex LH-20 (GE Healthcare Bio Sciences AB, Uppsala, Sweden), and MCI-gel CHP 20P (Mitsubishi Chemical Corp., Tokyo Met, Japan). All other chemicals were of analytical reagent grade and used without further purification.

### Plant materials

The buds of *D. genkwa* were collected from Bozhou, Anhui Province, China and were authenticated by Prof. Zhili Zhao of Shanghai University of Traditional Chinese Medicine, Institute of Chinese Materia Medica.

### Extraction and isolation

The dried flower buds of *D. genkwa* (20.0 kg) were extracted with 95% ethanol extensively at room temperature and evaporated to dryness to yield a dark crude ethanol extract (2 kg). The extract was dissolved in water to form a suspension and partitioned with petroleum ether (20 L×3), dichloromethane (20 L×3), *n*-butanol (20 L×3) successively and then evaporated under reduced pressure at 60°C to afford the petroleum fraction (346 g), dichloromethane fraction (277 g), n-butanol fraction (532 g), and water fraction (489 g). The extract was subjected to column chromatography with petroleum ether containing increasing amounts of acetic ether using a silica gel column to obtain aurantiamide acetate (**9**, 30 mg) and 10 fractions (Fr. 1–10). Fr. 3 was separated by MCI gel (CHP-20P) column chromatography using gradient elution from methanol/water, 30:70, to methanol/water, 100:0, to give genkwanin (**2**, 60 mg), 3′-hydroxygenkwanin (**3**, 35 mg) and 5 fractions (Fr. A-E). Fr. B and C were purified by chromatography over silica gel column using dichloromethane-methanol (9:1) to yield pinoresinol (**6**, 15 mg), daphnoretin (**1**, 8 mg) and syringaresinol (**7**, 20 mg). Fr. 5 was separated by column chromatography on silica gel eluting with dichloromethane-methanol (8:1) to give apigenin (**4,** 27 mg) and 3 fractions (Fr. F-H). Fr. G was further purified by chromatography over Sephadex LH-20 column eluting with methanol to give yuanhuacine (**10**, 19 mg), yuanhuadine (**11**, 23 mg). Fr. 6 was separated by column chromatography on MCI gel (CHP-20P) and Sephadex LH-20 using gradient elution from methanol/water, 30:70, to methanol/water, 100:0, to give tiliroside (**5**, 10 mg), pinoresinol (**6**, 10 mg) and genkwanine VIII (**13**, 11 mg). Fr. 7 was purified by column chromatography over Sephadex LH-20 eluting with methanol to give lariciresinol (**8**, 10 mg).

### Spectroscopic data

The NMR Data of Genkwanin VIII and the known compounds were in the Table 2 and Additional file 1.

### Cell lines and culture

MDA-MB-231 (breast cancer cell line) was cultured in DMEM supplemented with 10% FBS (fetal bovine serum), 2 mM glutamine, 100 units/mL penicillin, 100 μg/mL streptomycin, 100 μg/mL neomycin at 37°C in an incubator containing 5% CO_2_.

Huh7 cells (human hepatocellular carcinoma cells; ATCC, Bethesda, MD) were grown in 90% DMEM supplemented with 10% FBS, 2 mM glutamine, 1.5 g/L sodium bicarbonate, 0.1 mM non-essential amino acids, 1.0 mM sodium pyruvate, 100 units/mL of penicillin, and 100 μg/mL of streptomycin.

AGS cells (human gastric cancer cells) and Colo 205 cells (human colorectal carcinoma cells; ATCC, Bethesda, MD) were cultured in RPMI 1640 medium supplemented with 10% FBS, 100 units/mL of penicillin, and 100 μg/mL of streptomycin at 37°C in an incubator containing 5% CO_2_.

MCF-7, HT-29 cells, and B 16 cells were cultured in DMEM medium supplemented with 10% FBS, 100 units/mL of penicillin, and 100 μg/mL of streptomycin at 37°C in an incubator containing 5% CO_2_.

A549 cells (human non-small-cell lung cancer cell line) were cultured in RPMI 1640 supplemented with 10% FBS, 100 units/mL of penicillin, 100 μg/mL of streptomycin, G sodium, and 250 ng/mL of amphothericin B at 37°C in an incubator containing 5% CO_2_.

A2058 was cultured in DMEM medium supplemented with 10% FBS and 1% glutamine at 37 C in an incubator containing 5% CO_2_.

### MTT assay

All of the tested extracts and compounds were dissolved in DMSO and subsequently diluted in the culture medium before the cultured cells were treated. The cells were seeded in 24-well plates at a density. After 24 h, the culture medium was replaced with 1000 μL of serial dilution (0 μg to 20 μg/0 μg to 50 μg) of samples, and the solvent was incubated for 24 h. The final concentration of the solvent was <0.1% in the cell culture medium. MTT (400 μL of 0.5 mg/mL) in PBS was added to each well. After incubation at 37°C for 4 h, the unreacted dye was removed, an equal cell culture volume of 10% SDS (400 μL) was added to dissolve the MTT formazan, and the absorbance was determined at 570 nm (A570). Cell viability (%) was calculated as follows: (A570 of treated cells/A570 of untreated cells) × 100.

### Experimental animals

Kunming mice weighing 18 g to 22 g with equal numbers of males and females were purchased from Shanghai SLAC Laboratory Animal Co., Ltd. These animals were used in accordance with the PR China legislation on the use and care of laboratory animals and approved by the Experimental Animal Ethical Committee of Shanghai University of Traditional Chinese Medicine. Murine sarcoma S180 cell line was obtained from SLAC Laboratory and reproduced in our laboratory.

### Preparation of S180 tumor-bearing mice

Mouse sarcoma S180 cell line was harvested and the cells were resuspended in NS at 1.0 × 10^7^ cells/mL to 1.3 × 10^7^ cells/mL. Fifty Kunming mice weighing 18 g to 22 g with equal numbers of males and females were used and subcutaneously implanted with 1.0 mL/mouse on the right flank. At 24 h after inoculation, the mice were randomly divided in five groups. 5-Fu-treated mice were injected intraperitoneally with 5-Fu as a positive drug at a dose of 25 mg/kg once every other day. Normal and control (tumor-inoculated) group of mice received a daily oral administration of 0.5% CMC-Na (0.2 mL/kg). The treated groups received the indicated concentrations of dichloromethane fraction (20 mg/mL to 700 mg/mL) by intragastric administration for 14 d starting from 24 h after tumor inoculation. At 15 d, all of the mice were sacrificed. The tumors were excised, weighed, and photographed.

### Western blot analysis

Approximately 1 × 10^6^ MCF-7 cells per 60 mm dish were grown in DMEM medium supplemented with 10% FBS, 100 units/mL of penicillin, and 100 μg/mL of streptomycin to form a nearly confluent monolayer. The cells were incubated with a maximum of 20 μg/mL of yuanhuacine for 24 h, detached and washed once in cold PBS, and resuspended in 100 μL of lysis buffer [10 mM Tris–HCl (pH 8), 0.32 M sucrose, 1% Triton X-100, 5 mM EDTA, 2 mM dithiothreitol, and 1 mM phenylmethyl sulfonyl fluoride]. The suspension was placed in an ice bath for 20 min and then centrifuged at 16,000 × rpm for 20 min at 4°C. Total protein content was determined using a Bio-Rad protein assay reagent with bovine serum albumin as the standard. Protein extracts were reconstituted in a sample buffer [0.062 M Tris–HCl, 2% sodium dodecyl sulfate (SDS), 10% glycerol, and 5% β-mercaptoethanol]. The mixture was then boiled for 5 min. Equal amounts of the denatured proteins were loaded into each lane and separated on 8% to 15% SDS polyacrylamide gels. Afterward, the proteins were transferred to polyvinylidene difluoride membranes overnight. These membranes were blocked with 0.1% Tween-20 in PBS containing 5% non-fat dried milk for 20 min at room temperature, allowed to react with primary antibodies for 2 h, and incubated with a horseradish peroxidase-conjugated goat anti-rabbit or anti-mouse antibody for 2 h before these membranes were developed using SuperSignal ULTRA chemiluminescence substrate (Pierce, Rockford, IL). Band intensities were quantified by densitometry at an absorbance of 540 nm determined using an enzyme-linked immunosorbent assay plate reader.

### Flow cytometric analysis

Cellular DNA content was determined by flow cytometric analysis of propidium iodide (PI)-labeled cells. MCF-7 cells (4 × 10^5^ cell/60 mm dish) were incubated with yuanhuacine (0, 5, 15, and 20 μg/mL) for 24 h. The cells were then collected by trypsinization and fixed in ice-cold 70% ethanol at -20°C overnight. The cells were resuspended in PBS containing 1% Triton X-100, 0.5 mg/mL of RNase and 4 μg/mL of PI at 37°C for 30 min. An FACS Calibur flow cytometer equipped with a single argon-ion laser (488 nm) was used for flow cytometric analysis. The cell cycle was determined and analyzed using Mod Fit software.

### Statistical analysis

Statistical analyses were performed in SPSS 11.0 and the data were analyzed using one-way ANOVA. The mean separations were performed using the least significant difference method. Each experiment was performed in triplicate, and all of the experiments were run thrice and yielded similar results. Measurements from all the replicates were combined and the treatment effects were analyzed.

## Competing interests

All the authors declare that they have no competing interests.

## Authors’ contributions

Simeng Li performed the experiments, analyzed the data and wrote the paper. Guixin Chou planned, analyzed the data and wrote the paper, You-Cheng Hseu and Hsin-Ling Yang planned the experiments. Zhiling Yu analyzed the data and wrote the paper. All authors read and approved the final manuscript.

## Supplementary Material

Additional file 1 Spectra report of compound 1-13. Click here for file
