# Supplementary material for: Isolation of anticancer constituents from flos genkwa (Daphne genkwa Sieb.et Zucc.) through bioassay-guided procedures
Source: Chem Cent J. 2013 Sep 23;7:159. doi: 10.1186/1752-153X-7-159 (PMC3851481; doi:10.1186/1752-153X-7-159)
Supplement: Additional file 1 — Spectra report of compound 1-13. [file 1752-153X-7-159-S1.docx]

**Supporting information**

Isolation of anticancer constituents from Flos Genkwa (***Daphne genkwa* Sieb.et Zucc.**) through bioassay-guided procedures

Simeng Li^a,b^, Guixin Chou^a,d,*^, Youcheng Hseu^b^, Hsinling Yang^c^, HiuYee Kwan^e^, Zhiling Yu^e,*^

**Abstract**

**Background:** Flos Genkwa (yuanhua in Chinese), the dried flower buds of *Daphne genkwa* Sieb.et Zucc. (Thymelaeaceae), is a traditional Chinese medicinal herb mainly used for diuretic, antitussive, expectorant, and anticancer effects. However, systematic and comprehensive studies on Flos Genkwa and its bioactivity are limited.

**Results:** After confirmation of the anti-tumor activity, the 95% ethanolic extract was subjected to successive solvent partitioning to petroleum ether, dichloromethane, n-butanol, and water soluble fractions. Each fraction was tested using the same biological activity model, and the dichloromethane fraction had the highest activity. The dichloromethane fraction was subjected to further chromatographic separation for the isolation of compounds **1–13**. Among the 13 compounds, the diterpene esters (compounds **10**–**13**) showed anticancer activity, whereas the flavonoids, lignanoids, and peptides showed moderate activity. Compound **13** was a new daphnane diterpenoid, which was named genkwanin VIII.

The preliminary antitumor mechanism of yuanhuacine was studied by protein expression and cell cycle analysis in MCF-7 cancer cells.

**Conclusion:** The present investigation tends to support the traditional use of Flos Genkwa for treating cancer. Through bioassay-guided fractionation and isolation techniques, the CH_2_Cl_2_ fraction was determined as the active fraction of the flower buds of *D. genkwa*, and the anti-tumor activity was ascribable to the compounds **10–13**.

**Keywords:** *Daphne genkwa*, Antitumor, MTT, Flow cytometric, Western blot.

Supporting material：

Figure **1**: HR-ESI-MS of compound **13**

Figure **2**: ^1^H-NMR of compound **13**

Figure **3**: ^13^C-NMR of compound **13**

Figure **4**: HSQC of compound **13**

Figure **5**: HMBC of compound **13**

Figure **6**: NOESY of compound **13**

Figure **7**: ^1^H-NMR of compound **1**

Figure **8**: ^13^C-NMR of compound **1**

Figure **9**: ^1^H-NMR of compound **2**

Figure **10**: ^13^C-NMR of compound **2**

Figure **11**: ^1^H-NMR of compound **3**

Figure **12**: ^13^C-NMR of compound **3**

Figure **13**: ^1^H-NMR of compound **4**

Figure **14**: ^13^C-NMR of compound **4**

Figure **15**: ^1^H-NMR of compound **5**

Figure **16**: ^13^C-NMR of compound **5**

Figure **17**: ^1^H-NMR of compound **6**

Figure **18**: ^13^C-NMR of compound **6**

Figure **19**: ^1^H-NMR of compound **7**

Figure **20**: ^13^C-NMR of compound **7**

Figure **21**: ^1^H-NMR of compound **8**

Figure **22**: ^13^C-NMR of compound **8**

Figure **23**: ^1^H-NMR of compound **9**

Figure **24**: ^13^C-NMR of compound **9**

Figure **25**: ^1^H-NMR of compound **10**

Figure **26**: ^13^C-NMR of compound **10**

Figure **27**: ^1^H-NMR of compound **11**

Figure **28**: ^13^C-NMR of compound **11**

Figure **29**: ^1^H-NMR of compound **12**

Figure **30**: ^13^C-NMR of compound **12**

Figure **1**: HR-ESI-MS of compound **13**

**
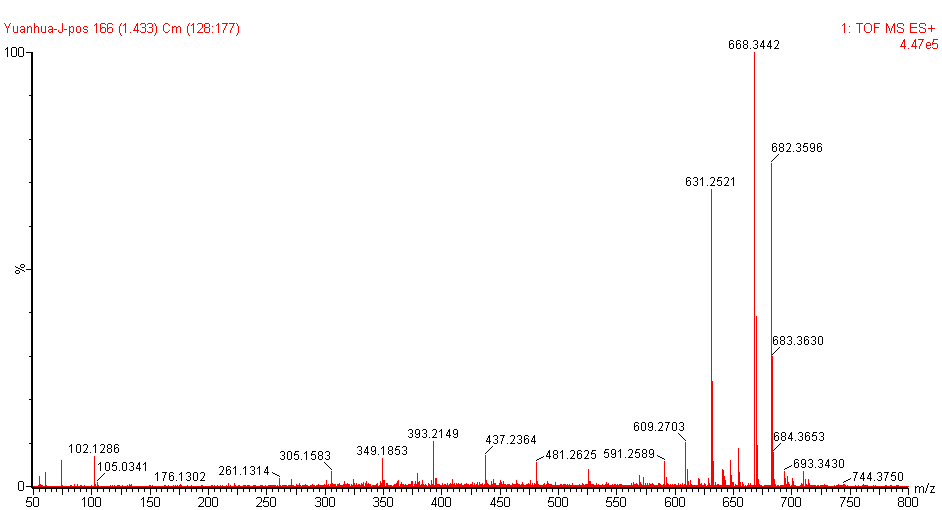
**

Figure **2**: ^1^H-NMR of compound **13**

Figure **3**: ^13^C-NMR of compound **13**

Figure **4**: HSQC of compound **13**

Figure **5**: HMBC of compound **13**

Figure **6**: NOESY of compound **13**

Figure **7**: ^1^H-NMR of compound **1**


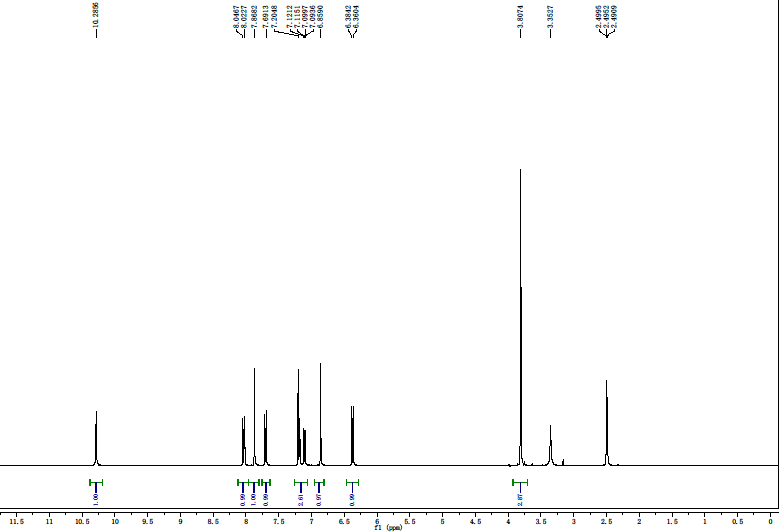


Figure **8**: ^13^C-NMR of compound **1**

**
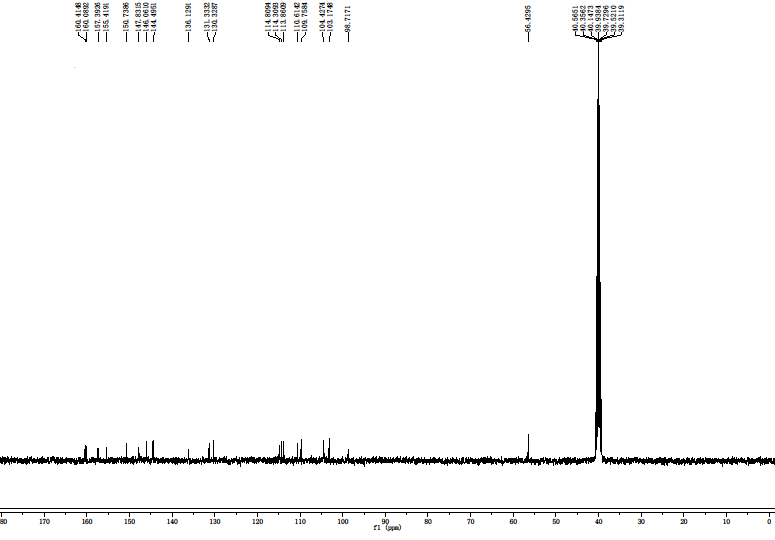
**

Figure **9**: ^1^H-NMR of compound **2**

**
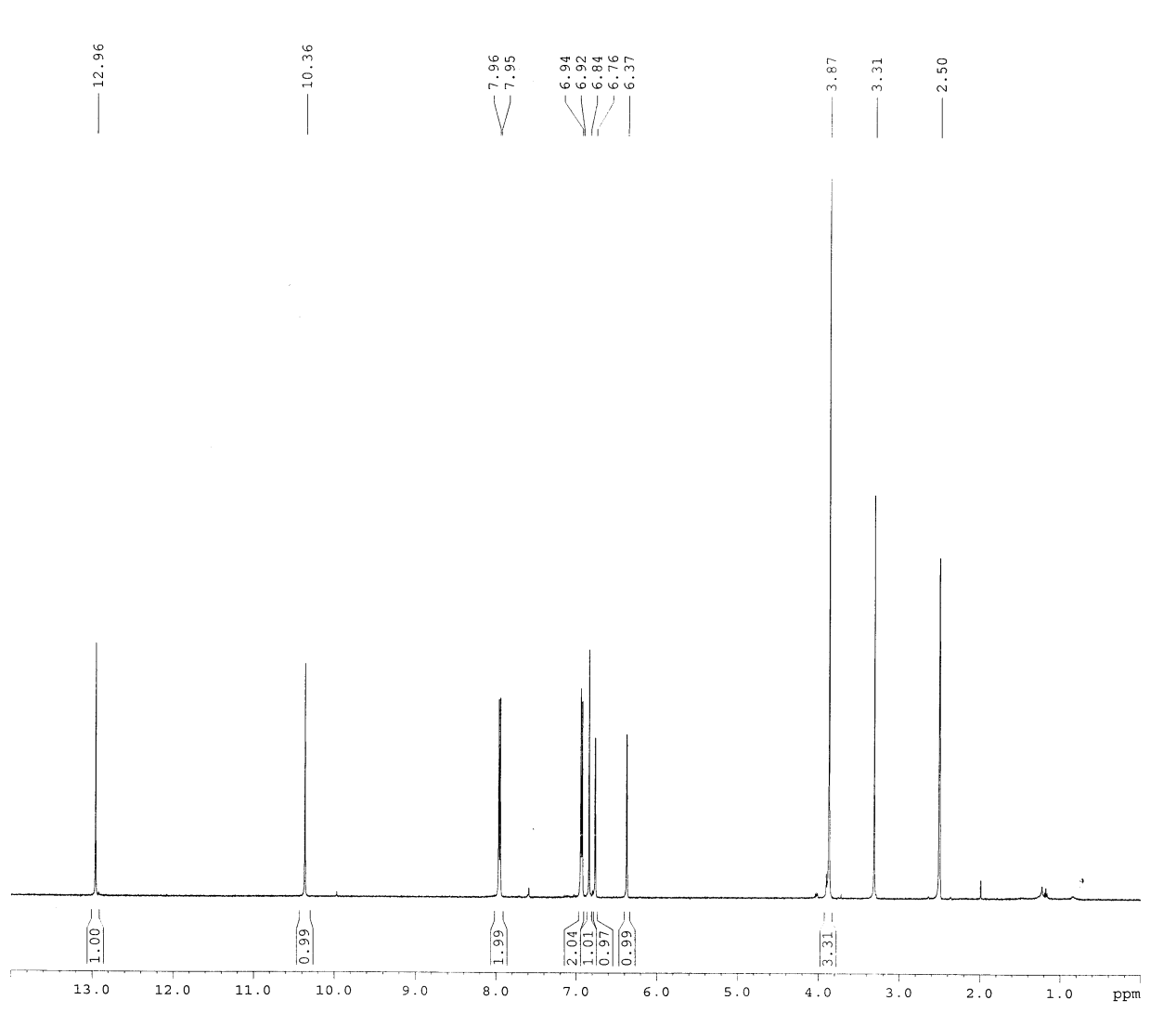
**

Figure **10**: ^13^C-NMR of compound **2**

Figure **11**: ^1^H-NMR of compound **3**

Figure **12**: ^13^C-NMR of compound **3**

Figure **13**: ^1^H-NMR of compound **4**

**
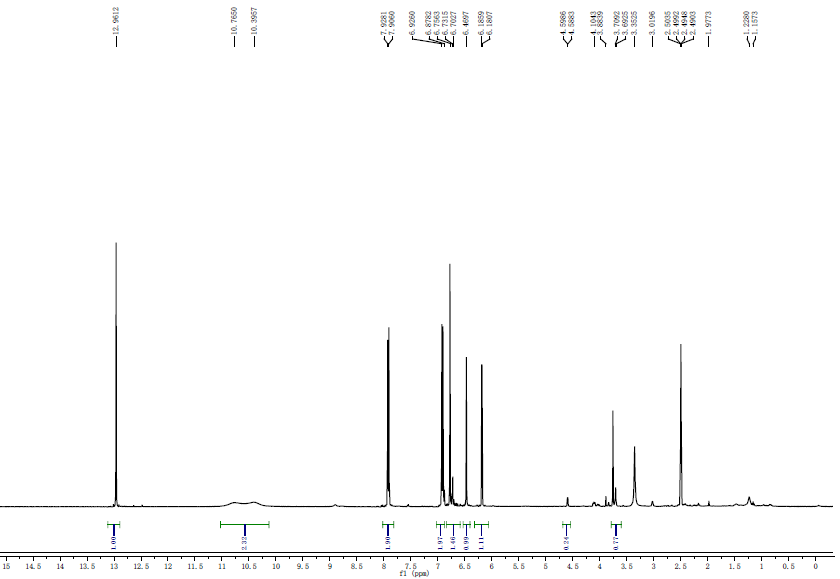
**

Figure **14**: ^13^C-NMR of compound **4**

**
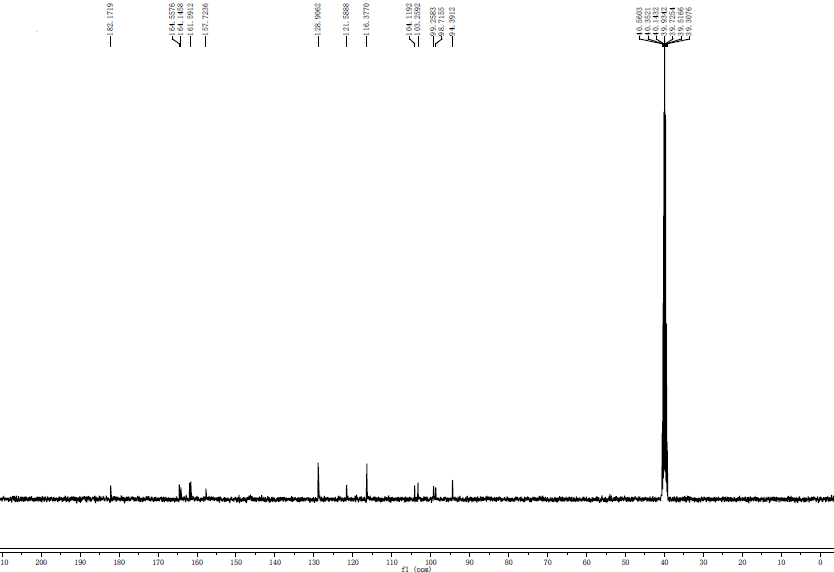
**

Figure **15**: ^1^H-NMR of compound **5**

Figure **16**: ^13^C-NMR of compound **5**

**
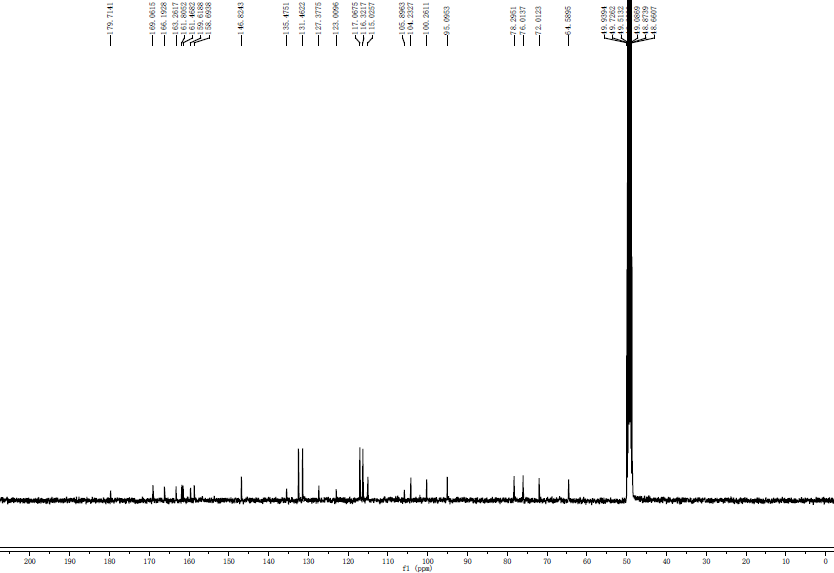
**

Figure **17**: ^1^H-NMR of compound **6**

**
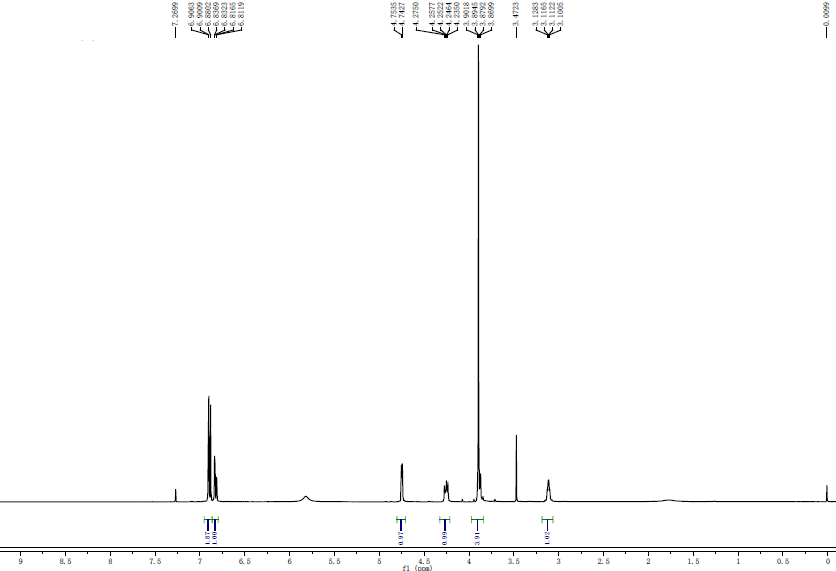
**

Figure **18**: ^13^C-NMR of compound **6**

**
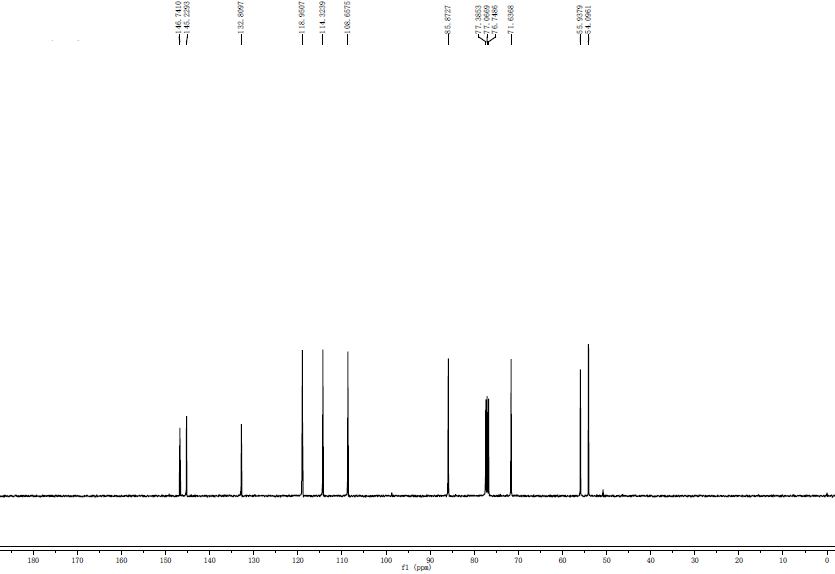
**

Figure **19**: ^1^H-NMR of compound **7**

Figure **20**: ^13^C-NMR of compound **7**

**
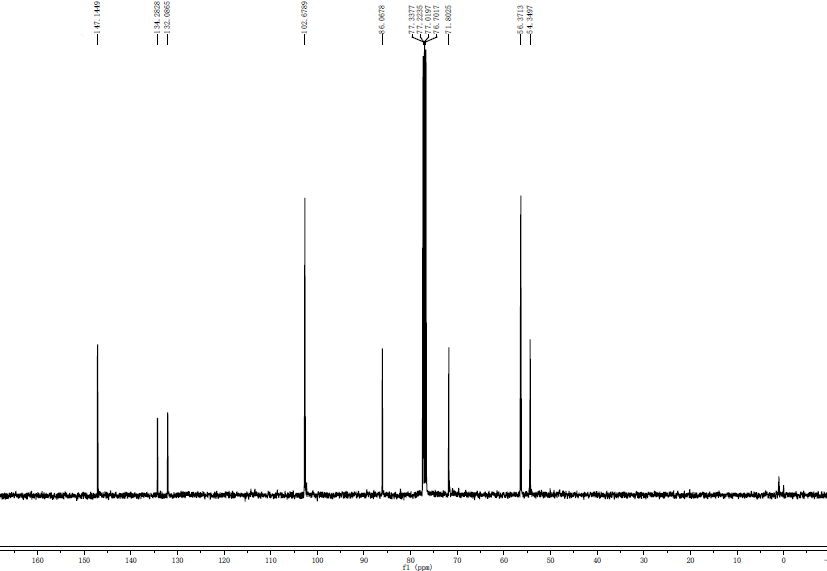
**

Figure **21**: ^1^H-NMR of compound **8**

Figure **22**: ^13^C-NMR of compound **8**

**
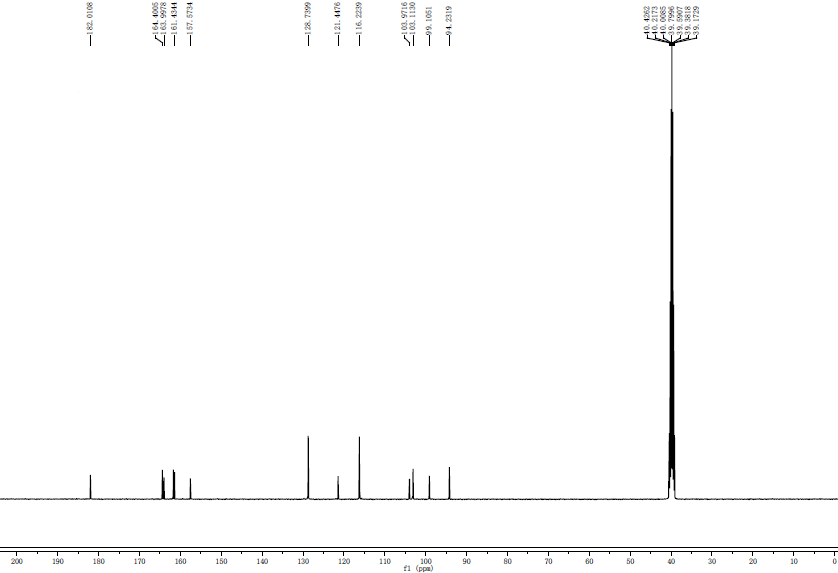
**

Figure **23**: ^1^H-NMR of compound **9**

Figure **24**: ^13^C-NMR of compound **9**

Figure **25**: ^1^H-NMR of compound **10**

Figure **26**: ^13^C-NMR of compound **10**

Figure **27**: ^1^H-NMR of compound **11**

Figure **28**: ^13^C-NMR of compound **11**

Figure **29**: ^1^H-NMR of compound **12**

Figure **30**: ^13^C-NMR of compound **12**
